# Anatomical Factors Influencing Furcation Regeneration: The Root Trunk. A Retrospective, Multicenter Clinical Study

**DOI:** 10.1002/cre2.70510

**Published:** 2026-11-09

**Authors:** Enrico Limiroli, Andrea Calò, Parham Hazrati, Pierpaolo Cortellini, Peter Eickholz, Akihiko Katayama, Jad Majzoub, Pamela McClain, Giulio Rasperini

**Affiliations:** ^1^ Department of Biomedical Surgical and Dental Sciences, University of Milan Milan Italy; ^2^ Foundation IRCCS Ca' Granda Polyclinic Via Della Commenda 10 Milan Italy; ^3^ Department of Periodontics and Oral Medicine University of Michigan, Ann Arbor Michigan USA; ^4^ Private Practice Florence Italy; ^5^ Department of Periodontology, Center for Dentistry and Oral Medicine (Carolinum) Johann Wolfgang Goethe‐University Frankfurt Am Main, 60596 Frankfurt Am Main Germany; ^6^ Private Practice Tokyo Japan; ^7^ Private Practice Aurora Colorado USA

## Abstract

**Objectives:**

The relationship between the anatomy of the root trunk and success in regenerative therapy of furcation defects is discussed in this article. The goal of this retrospective, multicenter clinical study is to clinically evaluate the relationship between the root trunk conformation and regenerative therapy success.

**Material and Methods:**

One hundred thirteen radiographs of mandibular molars with furcation defects that had been treated with regenerative therapy were collected from six clinical centers. Data on the type of therapy and clinical parameters before and after treatment (follow‐up of at least 12 months) were collected. The radiographs (before surgery and at least 12 months postoperatively) were measured with a visual evaluation method by a blind operator using graphics software.

**Results:**

Success was defined as: reduction in horizontal and vertical furcation involvement, decrease in probing depths, and increase in clinical attachment level. It was statistically assessed on 113 regenerated molar sites and related to root trunk. Also clinical variables such as age, sex, and treatment were evaluated. No correlation was found between success in regenerative therapy and the conformation of the root trunk.

**Conclusions:**

The regenerative outcome was not significantly influenced by the root trunk conformation. The center, the degree of furcation involvement, sex, and treatment were significantly associated with higher success of periodontal regeneration.

## 1 Introduction

In the realm of periodontal therapy, dental furcation regeneration emerges as a critical frontier, embodying the pursuit of restoring the structural and functional integrity of the periodontium. In literature, the efficacy of periodontal regeneration has been demonstrated through the use of biologics (Tavelli et al. 2022), guided tissue regeneration techniques (GTR) (Jepsen et al. 2002), and the use of bone graft (Swami et al. 2021). Furcation involvement, characterized by the loss of supporting bone and soft tissue in the interradicular space, poses a significant challenge in the management of periodontal diseases. Addressing furcation defects effectively not only enhances the stability of the affected teeth but also preserves the overall periodontal health and function. The anatomy of the interradicular space and the height of the root trunk are supposed to be fundamental factors in regeneration success, but literature is poor. Previous studies (Limiroli et al. 2023a; Limiroli et al. 2023b) findings were that the outcome of periodontal regeneration of furcation defects was not affected by the interradicular anatomy. The interradicular anatomy represented in the articles, however, while it is an expression of divergence and represents the anatomy of entry to the furcation, does not include additional anatomical parameters that are possibly related to success in regenerative therapy. The most significant, as said, isnt the root trunk, which is the area between the cemento–enamel junction (CEJ) and the point where the roots separate. We know that molars with short root trunks are generally at a higher risk for furcation involvement compared to those with longer trunks (Al‐Shammari et al. 2001; Hou and Tsai 1997; Seshima et al. 2017). That said, the research on whether root trunk length actually dictates the success of regenerative therapy is inconsistent. Given this uncertainty, it's clear that this anatomical factor needs a more standardized evaluation when determining a tooth's prognosis. In 2022, Limiroli et al. proposed a new way to map the interradicular space by looking at the width of the furcation roof and the distance between roots (Limiroli et al. 2023). By using proportional parameters, the method effectively bypasses the common issue of radiographic magnification, ensuring precise and reproducible results for any clinician. To ensure objectivity and consistency, we adopted the same linear measurement and ratio‐based approach to determine how root trunk dimensions actually influence regenerative therapy. The aim of this research is to assess the prognostic value of the root trunk, through proportional linear measurements, investigating its specific impact on the success of periodontal regeneration in furcation defects, defined in this study as simultaneous CAL gain and furcation degree reduction.

## 2 Materials and Methods

### 2.1 Study Design

This is a multicenter, retrospective clinical study with the main goal of investigating a possible relationship between root trunk length and the clinical outcomes of regenerative therapy. The data were collected from six clinical centers. The root trunk was measured using periapical radiographs taken at baseline and at follow‐up at least 12 months later.

### 2.2 Inclusion Criteria

Radiographs were included in the present study only if they met the following inclusion criteria:

1. Radiographs taken with a paralleling technique.
2. Good image definition (root margins clearly distinguishable).
3. Complete view of the root trunk and central fossa of mandibular molar.
4. Infrabony defect of at least 3 mm.
5. Absence of endodontic lesions.
6. Interproximal bone level coronal or at the same level as the roof of the furcation.

### 2.3 Exclusion Criteria

Radiographs with extreme roots overlapping, incomplete view of interradicular space, or any other reason which made the images unreadable were excluded.

### 2.4 Data Collection

A total of 113 periapical radiographs of mandibular molars that met the inclusion criteria and had undergone regenerative treatment were gathered from six clinical sites. From each site, the following anonymized data were collected: (i) type of regenerative procedure performed; (ii) demographic details of patients, including age and gender; (iii) initial and final vertical probing depths (PD) at the midfacial aspect; (iv) depth of midfacial gingival recession (REC); (v) midfacial clinical attachment level (CAL), calculated by adding REC to PD; and (vi) horizontal and vertical furcation class at the last follow‐up, following the Hamp and Tonetti classification. Treatment success was defined as the presence of gain in clinical attachment level and simultaneous improvement in furcation involvement (horizontal and/or vertical reduction of furcation defect) compared to baseline.

A trained operator, blinded to the center, technique used, and eventual clinical results, conducted measurements on root trunk X‐rays. Given the retrospective nature of the study, individual centers were not standardized. Clinical data and X‐rays were forwarded to a central facility, where a calibrated operator analyzed the information and conducted measurements on the X‐rays. Essentially, the operator assessed the root trunks using graphic software (Paint, Windows10, Microsoft, USA) in this study. Two bi‐dimensional measure were calculated on which a ratio was calculated to overcome the error of magnification of bi‐dimensional images:

- CF: a vertical line from the central fossa of molars to the roof of the furcation (Figure 1).
- RT: a vertical line from the CEJ to the roof of the furcation (Figure 1).

**Figure 1:**
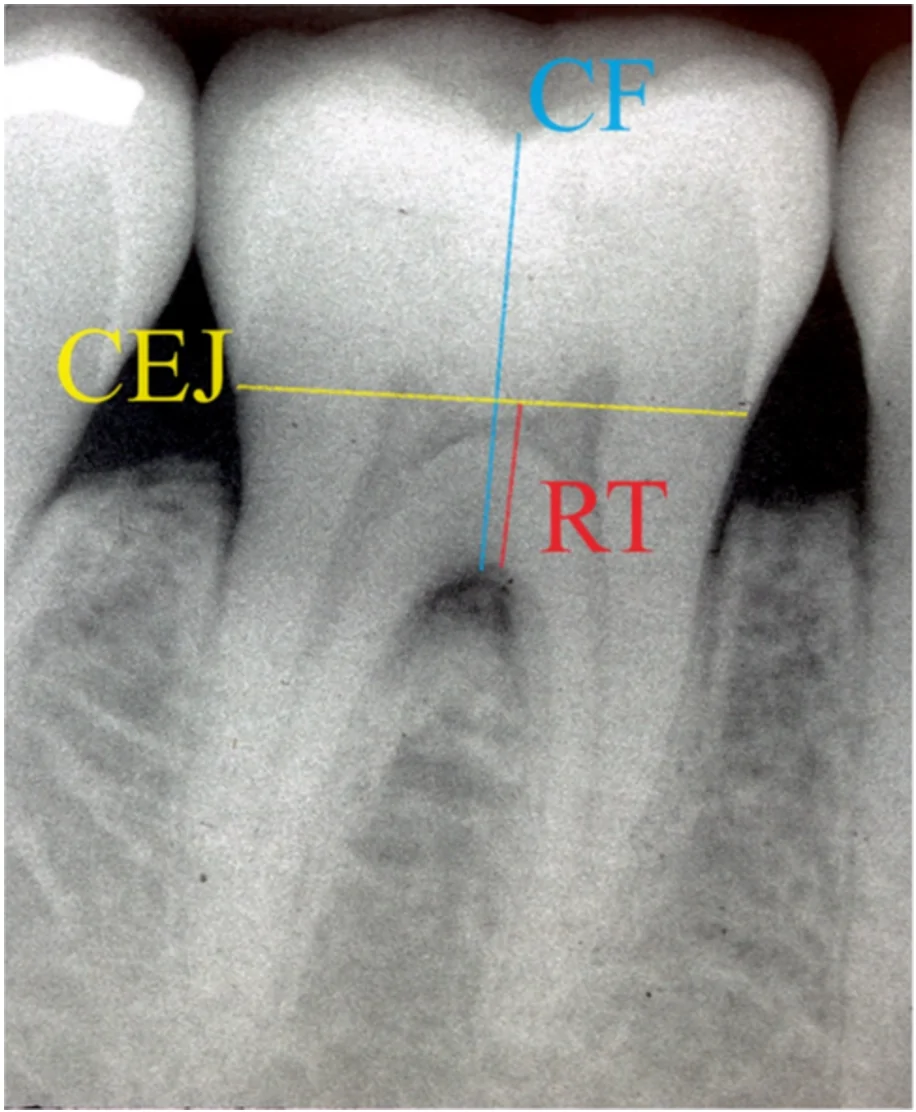
The root trunk ratio was calculated on CF/RT for each Peri‐apical X‐ray.

### 2.5 Statistical Analysis

The data were descriptively summarized using means and standard deviations (SD) for continuous variables and absolute and relative frequency for binary and categorical variables. Afterward, the statistical analysis proceeded in a stepwise manner. The primary outcome, treatment success (coded as “ok”), and secondary outcomes, including CAL gain (coded as CAL‐gain) and furcation degree reduction (coded as G‐red), were analyzed using univariable and multivariable mixed‐effects logistic regression models.

The univariable mixed‐effects model assessed the association between each dependent variable, including treatment success, CAL gain, and furcation degree reduction, and each of the following covariates individually: the Root Trunk Ratio of central pit‐furcation/root trunk (predictor), age, sex, treatment received (TRT), keratinized tissue width at baseline (kt1), horizontal furcation involvement at baseline (hf1), and vertical furcation involvement at baseline (vf1). In the next step, any covariate demonstrating an association with the dependent variable with a *p* value of less than 0.05 was included in the multivariable mixed‐effects model. In both univariable and multivariable models, a random intercept for center was included to account for the clustering effect imposed by different treatment centers.

In addition, mixed‐effects linear regression models were used to explore the linear relationship between the covariates and the continuous outcomes of horizontal and vertical furcation involvement reduction.

All statistical analyses were performed using RStudio (Version 2026.01.0 + 392, RStudio PBC) with packages “lme4”, “lmerTest”, “ggplot2”, “car”, and “performance.” Models were fitted using maximum likelihood estimation with the bobyqa optimizer to ensure convergence. Multicollinearity among covariates in the multivariable models was assessed using the Variance Inflation Factor (VIF), and all variables demonstrated low VIF values, indicating no significant multicollinearity. Model fit was evaluated using pseudo‐*R* ^2^ values for logistic models and *R* ^2^ measures for linear mixed‐effects, and all multivariable models demonstrated *R* ^2^ values between 0.5 and 0.8. Statistical significance was set at *p* < 0.05. The proposed statistical analyses addressed all study objectives.

## 3 Results

### 3.1 Study Population and Baseline Characteristics

A total of 138 mandibular molars from six clinical centers were included in this study. The study population had an average age of 49.66 ± 10.04 years, with a female prevalence of 61.54%. At baseline, the mean horizontal furcation involvement was 2.04 ± 0.40 mm, and the mean vertical involvement was 1.95 ± 0.69 mm. The mean width of keratinized tissue was 2.99 ± 1.17 mm. Detailed baseline characteristics for each center are summarized in Table 1.

**Table 1:** Baseline demographic and clinical characteristics of the study population.

	Subjects (*N*)	Females (*N)*/(%)	Age (mean ± SD) (years)	Baseline keratinized tissue width (mean ± SD) (mm)	Baseline horizontal furcation involvement (mean ± SD) (mm)	Baseline vertical furcation involvement (mean ± SD) (mm)
Center 1	23	11/47.83	53.83 ± 8.59	3.74 ± 1.14	2.22 ± 0.42	2.26 ± 0.81
Center 2	20	10/50	43.95 ± 10.65	NA	2.10 ± 0.64	1.15 ± 0.37
Center 3	15	14/93.33	47.80 ± 11.91	3.2 ± 1	2.00 ± 0.00	1.80 ± 0.68
Center 4	18	11/61.11	50.33 ± 10.62	2.44 ± 1.04	2.06 ± 0.24	2.22 ± 0.73
Center 5	15	10/66.67	51.93 ± 3.81	2.67 ± 0.8	2.20 ± 0.41	1.87 ± 0.52
Center 6	47	NA	NA	NA	1.89 ± 0.31	2.11 ± 0.48
Overall	138	56/61.54	49.66 ± 10.04	2.99 ± 1.17	2.04 ± 0.40	1.95 ± 0.69

### 3.2 Primary Outcome: Treatment Success

The multivariable mixed‐effects logistic regression analysis (Table 2) identified specific predictors for treatment success (defined as simultaneous CAL gain and furcation degree reduction).

- Root Trunk Ratio: The ratio between the central fossa‐furcation distance and the root trunk length did not significantly influence the primary outcome (*p* = 0.381).
- Gender: Male patients showed significantly lower odds of treatment success compared to females (OR = 0.20; 95% CI: [0.05, 0.84]; *p* = 0.028).
- Baseline Vertical Involvement (vf1): Higher initial vertical furcation depth was associated with significantly higher odds of success (OR = 6.29; 95% CI: [1.51, 26.20]; *p* = 0.012).
- Baseline Horizontal Involvement (hf1): This variable did not show a statistically significant association with overall success in the multivariable model (*p* = 0.191).

**Table 2:** Univariable and multivariable mixed‐effects logistic regression analyses of factors associated with treatment success.

~Success	Univariable mixed‐effects model					Multivariable mixed‐effects model			
Variable	OR	Lower	Upper	*p* value	Patients	OR	Lower	Upper	*p* value
Age	0.98	0.92	1.04	0.505	91				
Sex					91				
*Female (intercept)*	12.90	2.59	64.1	**0.002**					
Male	0.21	0.06	0.78	**0.020**		0.20	0.05	0.84	**0.028**
Treatment					120				
*GTR (intercept)*	1.93	0.43	8.72	0.394					
EMD	1.92	0.26	14.50	0.525					
FGF2	NA	NA	NA	NA					
Root Trunk Ratio	2.45	0.33	18.10	0.381	113				
Baseline keratinized tissue width	1.96	0.92	4.14	0.080	71				
Baseline horizontal furcation involvement	7.90	1.56	40.00	**0.012**	138	5.70	0.42	77.70	0.191
Baseline vertical furcation involvement	2.79	1.22	6.41	**0.015**	138	6.29	1.51	26.20	**0.011**

*Note:* Bold values indicate statistical significance.

### 3.3 Secondary Outcomes: CAL Gain and Furcation Reduction

Regarding CAL Gain (Table 3), root trunk ratio didn't reach statistical significance for this parameter. The multivariable model confirmed that the male gender was associated with a lower probability of achieving significant attachment gain (OR = 0.14; *p* = 0.020). No other covariates, reached statistical significance for this parameter.

**Table 3:** Univariable and multivariable mixed‐effects logistic regression analyses of factors associated with CAL Gain.

~CAL Gain	Univariable mixed‐effects model					Multivariable mixed‐effects model			
Variable	OR	Lower	Upper	*p* value	Patients	OR	Lower	Upper	*p* value
Age	0.96	0.89	1.04	0.354	91				
Sex					91				
*Female (intercept)*	46.60	4.52	481.11	**0.001**					**0.020**
Male	0.14	0.03	0.74	**0.020**		0.14	0.03	0.74	
Treatment					120				
*GTR (intercept)*	2.89	1.06	7.85	0.375					
EMD	5.18	0.77	34.90	0.091					
FGF2	NA	NA	NA	0.997					
Root Trunk Ratio	2.15	0.19	24.70	0.540	113				
Baseline keratinized tissue width	2.99	0.87	10.3	0.081	71				
Baseline horizontal furcation involvement	1.06	0.34	3.30	0.915	138				
Baseline vertical furcation involvement	1.00	0.37	2.72	0.996	138				

*Note:* Bold values indicate statistical significance.

Talking about Furcation Degree Reduction (Table 4), root trunk ratio didn't reach statistical significance. Both baseline horizontal (OR = 16.2; *p* = 0.013) and vertical involvement (OR = 4.15; *p* = 0.006) were strong predictors of improvement in the multivariable analysis.

**Table 4:** Univariable and multivariable mixed‐effects logistic regression analyses of factors associated with Furcation degree reduction.

~Furcation Degree Reduction	Univariable mixed‐effects model					Multivariable mixed‐effects model			
Variable	OR	Lower	Upper	*p* value	Patients	OR	Lower	Upper	*p* value
Age	0.96	0.89	1.04	0.354	91				
Sex					91				
*Female (intercept)*	12.2	3.74	40.10	< 0.001					
Male	0.32	0.09	1.15	0.080					
Treatment					120				
*GTR (intercept)*	3.38	0.99	11.50	0.052					
EMD	1.20	0.19	7.65	0.844					
FGF2	NA	NA	NA	0.999					
Root Trunk Ratio	1.51	0.28	8.17	0.634	113				
Baseline keratinized tissue width	1.96	0.92	4.14	0.081	71				
Baseline horizontal furcation involvement	18.20	2.10	157.00	**0.008**	138	16.2	1.78	147.63	**0.013**
Baseline vertical furcation involvement	4.82	1.75	13.20	**0.002**	138	4.15	1.51	11.40	**0.006**

*Note:* Bold values indicate statistical significance.

### 3.4 Linear Correlation and Furcation Reduction

Linear mixed‐effects models explored the continuous reduction of furcation defects (Baseline—Follow‐up). The analysis, visualized through bubble plots, revealed a direct proportional relationship (Figure 2 and 3):

**Figure 2:**
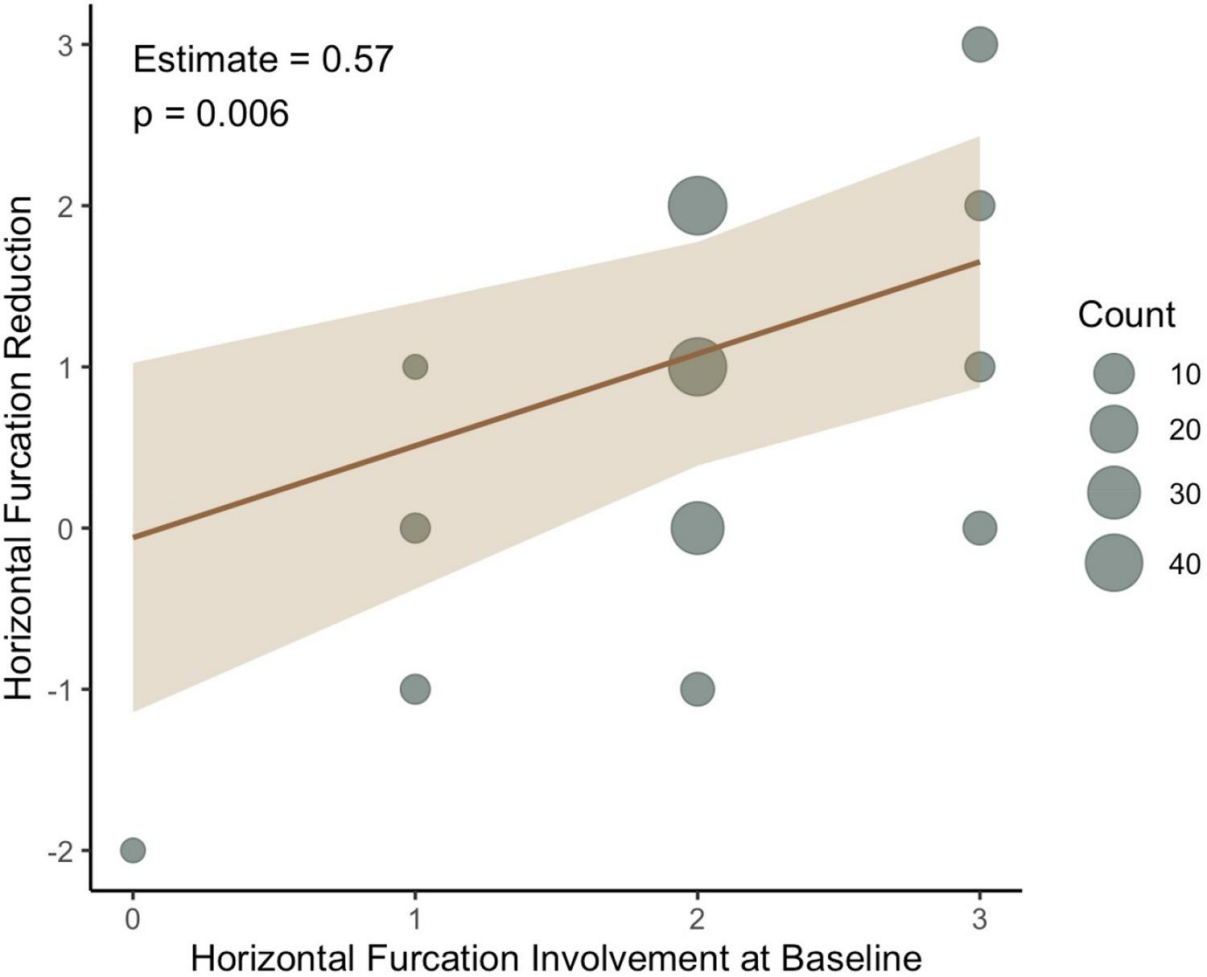
Bubble plot showing the relationship between baseline horizontal furcation involvement and horizontal furcation reduction at follow up. The *x*‐axis represents the baseline horizontal furcation involvement scored according to the Hamp classification (Grades 0 to 3). The *y*‐axis illustrates the horizontal furcation reduction achieved at follow‐up, representing the change in degrees (e.g., a positive value indicates clinical improvement, such as a score of +2 for a shift from Grade 3 to Grade 1; a negative value indicates disease progression, such as a score of −1 for a shift from Grade 1 to Grade 2). The size of the circles corresponds to the patient count for each observation. A statistically significant positive linear relationship is observed (Estimate = 0.57, *p* = 0.006), with the shaded area indicating the 95% confidence interval.

**Figure 3:**
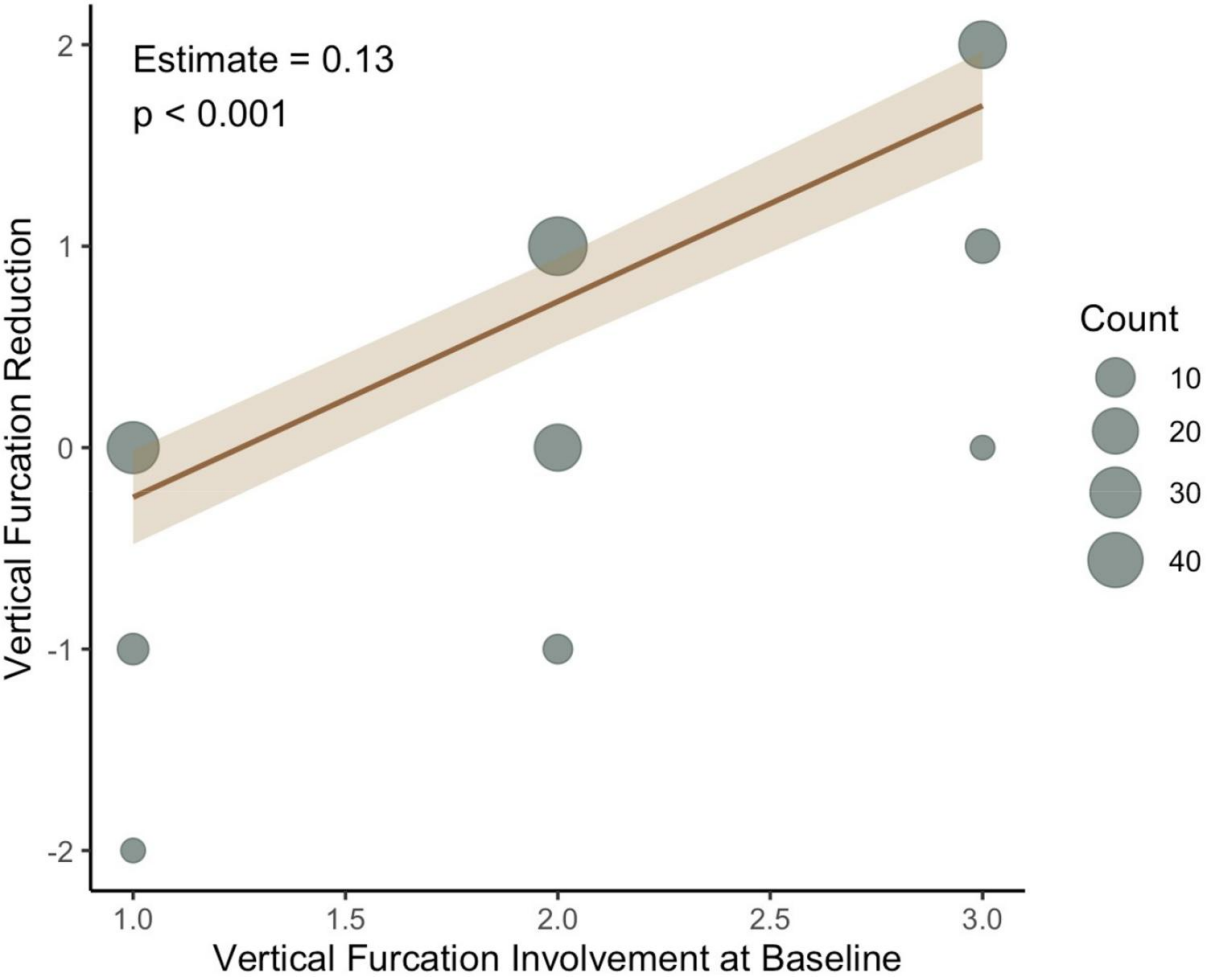
Bubble plot showing the relationship between baseline vertical furcation involvement and vertical furcation reduction at follow up. The *x*‐axis represents the baseline vertical furcation involvement scored according to the Tonetti classification, where grade A corresponds to 1.0, grade B to 2.0, and grade C to 3.0. The *y*‐axis illustrates the vertical furcation reduction achieved at follow‐up, representing the shift between classes (e.g., a positive value indicates improvement, such as a score of +2 for a shift from C to A, or +1 from C to B; a negative value indicates disease progression, such as a score of −1 for a shift from A to B, or −2 from A to C). The size of the circles corresponds to the patient count for each observation. A statistically significant positive linear relationship is observed (Estimate = 0.13, *p* < 0.001), with the shaded area indicating the 95% confidence interval.

Horizontal Reduction: A significant positive correlation was found with baseline involvement (Estimate = 0.57, *p* = 0.006) (Table 5).

**Table 5:** Univariable and multivariable mixed‐effects logistic regression analyses of factors associated with Horizontal furcation involvement.

~Horizontal Furcation Reduction (mm)	Univariable mixed‐effects model					Multivariable mixed‐effects model			
Variable	Estimate	Lower 95% CI	Upper 95% CI	*p* value	Patients	Estimate	Lower 95% CI	Upper 95% CI	*p* value
Age	−0.01	−0.03	0.01	0.440	91				
Sex					91				
*Female (intercept)*	1.55	1.01	2.10	0.00		−0.06	−1.18	1.06	0.91
Male	−0.59	−0.95	−0.23	1		−0.39	−0.77	−0.15	4
				**0.002**					**0.042**
Treatment					120				
*GTR (intercept)*	0.86	0.09	1.63	0.03		−0.06	−1.18	1.06	0.91
EMD	0.19	−0.48	0.86	5		0.13	−0.51	0.78	4
FGF2	1.65	0.65	2.65	0.574		1.40	0.44	2.36	0.676
				**0.002**					**0.006**
Root trunk ratio	0.14	−0.10	0.37	0.246	113				
Baseline keratinized tissue width	0.06	−0.12	0.25	0.494	71				
Baseline horizontal furcation involvement	0.76	0.41	1.12	**< 0.001**	138	0.57	0.17	0.97	**0.006**
Baseline vertical furcation involvement	0.17	−0.07	0.41	0.174	138				

*Note:* Bold values indicate statistical significance.

Vertical Reduction: Similarly, higher baseline vertical involvement led to a greater absolute reduction in depth (Estimate = 0.13, *p* < 0.001) (Table 6).

**Table 6:** Univariable and multivariable mixed‐effects logistic regression analyses of factors associated with Vertical furcation involvement.

~Vertical Furcation Reduction (mm)	Univariable mixed‐effects model					Multivariable mixed‐effects model			
Variable	Estimate	Lower 95% CI	Upper 95% CI	*p* value	Patients	Estimate	Lower 95% CI	Upper 95% CI	*p* value
Age	−0.01	−0.02	0.01	0.449	91				
Sex					91				
*Female (intercept)*	0.74	0.19	1.30	0.019					
Male	0.06	−0.26	0.37	0.724					
Treatment					120				
*GTR (intercept)*	0.47	−0.13	1.07	0.090					
EMD	0.20	−0.36	0.77	0.451					
FGF2	0.66	−0.17	1.51	0.111					
Root trunk ratio	0.13	−0.07	0.33	0.202	113				
Baseline keratinized tissue width	0.02	−0.15	0.19	0.828	71				
Baseline horizontal furcation involvement	0.43	0.10	0.75	**0.011**	138	0.13	−0.07	0.34	0.203
Baseline vertical furcation involvement	0.99	0.86	1.12	**< 0.001**	138	0.97	0.84	1.10	**< 0.001**

*Note:* Bold values indicate statistical significance.

## 4 Discussion

The present multicenter retrospective study aimed to clarify the role of the root trunk, quantified through a reproducible radiographic ratio (Root Trunk Ratio), on the outcomes of regenerative therapy in mandibular furcation defects. Contrary to some previous anatomical assumptions (Al‐Shammari et al. 2001; Hou and Tsai 1997), our findings suggest that the root trunk dimension does not significantly dictate the clinical success of regenerative procedures.

The introduction of the CF/RT ratio was intended to bypass radiographic magnification errors, providing a standardized anatomical map. While previous literature Hou and Tsai 1997) suggested that shorter root trunks might lead to earlier furcation involvement, our data indicate that once the defect is established and treated regeneratively, the length of the trunk itself does not limit the potential for attachment gain or bone fill. This aligns with the findings of Limiroli et al. (2023), reinforcing the idea that interradicular anatomy, while complex, does not affect regenerative predictability, by specifically addressing the root trunk, a parameter previously considered a potential prognostic determinant.

A key finding of this study is the significant influence of baseline defect severity on the magnitude of the regenerative response. The multivariable analysis and linear models (Figure 2 and 3) clearly demonstrate that deeper defects (both horizontal and vertical) achieve greater absolute reductions. Specifically, a high baseline vertical involvement (vf1) was associated with a sixfold increase in the odds of treatment success (OR = 6.29). This “potential for improvement” suggests that regenerative therapies are particularly effective in more advanced Class II defects, where the space‐providing capacity of the defect morphology might favor clot stability and graft containment. It is consistent with similar findings in intrabony defects, which typically exhibit more regenerative potential with deeper defect anatomy (Seshima et al. 2017; Bowers et al. 2003; Horwitz et al. 2004).

Interestingly, gender emerged as a significant secondary predictor, with males showing lower odds of success and CAL gain. While the biological mechanism remains speculative, this could be related to differences in healing potential, bone density, or compliance, warranting further investigation in prospective cohorts.

Moreover, the lack of significant association between the treatment type (GTR, EMD, FGF2) and success suggests that the biological potential of the site and patient‐specific factors may outweigh the specific regenerative material used. However, the retrospective nature of this study, the small sample, and possibly the lack of standardization across the six centers represent a limitation.

## 5 Conclusion

Within the limitation of the present study, the following conclusion can be obtained. The root trunk dimension, quantified through the standardized CF/RT ratio, does not significantly influence the clinical outcomes of regenerative therapy in mandibular furcation defects and is not to be considered a predictional factor in furcation regeneration. Baseline defect severity emerged as the strongest predictor of regenerative success. Greater vertical furcation involvement at baseline was associated with treatment success due to favorable wound stability and space‐providing morphology (Nibali et al. 2021). No significant differences in regenerative outcomes were observed among the treatment modalities employed (GTR, EMD, FGF‐2).

Considering the previous study about root divergence and our conclusions about root trunk, it begins to emerge more clearly that a “single” anatomical factor will unlikely be the critical factor in furcation regeneration success and that site biology and patient‐specific factors may play a more determinant role, creating a combination of complex factors that contribute to positive long‐term outcomes.

Future studies should aim to validate these findings in larger, multicenter cohorts and explore the biological mechanisms underlying gender‐anatomy‐related differences in healing. In doing so, we can refine patient selection criteria and improve the predictability of regenerative periodontal therapies.

## Author Contributions

Enrico Limiroli, Andrea Calò, Giulio Rasperini, Parham Hazrati conceived and designed the study. All the authors have been involved in data collection. Enrico Limiroli, Andrea Calò, Giulio Rasperini, and Parham Hazrati have been involved in data interpretation, drafting the manuscript and revising it critically. The authors have given final approval of the version to be published.

## Ethics Statement

The study protocol was approved by the ethics committee of the University of Milan, Italy (28/04/2022).

## Conflicts of Interest

The authors declare no conflicts of interest.

## Data Availability

The data that support the findings of this study are available on request from the corresponding author. The data are not publicly available due to privacy or ethical restrictions.
